# Histomorphometric characterization of the uterus and placenta in Piau and Commercial sows during early gestation

**DOI:** 10.1590/1984-3143-AR2025-0070

**Published:** 2026-01-26

**Authors:** Tânia Fernandes Martins, Lívia Maria dos Reis Barbosa, Luiz Otávio Guimarães Ervilha, Mariana Machado Neves, Alysson Saraiva, José Domingos Guimarães, Mateus Guimarães dos Santos, Paula da Fonseca Pereira, Simone Eliza Facioni Guimarães

**Affiliations:** 1 Programa de Pós-graduação em Zootecnia, Universidade Federal de Viçosa, Viçosa, MG, Brasil; 2 Archer Daniels Midland – ADM Brasil, São Paulo, SP, Brasil; 3 Programa de Pós-graduação em Biologia Celular e Estrutural, Universidade Federal de Viçosa, Viçosa, MG, Brasil; 4 Programa de Pós-graduação em Medicina Veterinária, Universidade Federal de Viçosa, Viçosa, MG, Brasil; 5 Laboratório de Biotecnologia Animal, Departamento de Zootecnia, Universidade Federal de Viçosa, Viçosa, MG, Brasil

**Keywords:** fetal development, endometrium, morphometry, placental vascularization, pig

## Abstract

Pregnancy induces critical physiological adaptations to support embryonic development and fetal survival. This study compared endometrial and placental phenotypic and histomorphometric characteristics of Piau and Commercial sows at two gestational ages (25 and 35 days). Twelve sows (six Piau and six Commercial) were evaluated in a randomized design, with samples collected from three regions of the right uterine horn of each animal. Histomorphometric analyses were performed using microscopy and ImageJ software. Statistical analyses employed linear mixed-effects models, with Shapiro-Wilk and Levene’s tests applied to assess normality and homogeneity of variances, respectively. At 25 days of gestation, Commercial sows showed greater uterine and ovarian weights, a higher number of corpora lutea, and longer uterine horn horns, reflecting genetic selection for reproductive efficiency. Conversely, Piau sows exhibited more advanced embryonic development at this stage, with fetuses of greater size. At 35 days, the phenotypic superiority of Commercial sows persisted, while Piau fetuses maintained greater weight and length, indicating distinct temporal growth dynamics. Histomorphometric analyses at 25 days revealed that Commercial sows had increased placental connective tissue deposition and thicker endometrial epithelium, whereas Piau sows presented larger placental vascular area, as well as enhanced endometrial vascularization and glandular density across all uterine regions. At 35 days, no significant differences were observed in placental vascular area and endometrial vascularization; however, subtle trends in connective tissue development suggested ongoing placental differentiation. These findings highlight distinct reproductive strategies between Piau and Commercial sows, with potential implications for embryonic development and gestational success. Altogether, the results confirm that genetic background influences uterine and placental morphology during early gestation.

## Introduction

Pregnancy involves maternal adaptations characterized by morphofunctional, metabolic, and hormonal changes in the uterus, particularly within the endometrium and placenta, essential for embryonic viability and proper placental formation (Almeida and Alvarenga Dias, 2022). To ensure pregnancy establishment and maintenance, the uterus undergoes significant structural and functional remodeling, mainly through angiogenesis and vasculogenesis, processes that are fundamental for vascular network development and adequate support for conceptuses growth and survival (Stenhouse et al., 2018).

Despite these adaptations, limitations in placental efficiency during early gestation remain a major cause of prenatal mortality, compromising embryonic viability (Kridli et al., 2016). In this context, the functional efficiency of the placenta, along with the adaptive capacity of the uterus, plays a crucial role in conceptuses survival and development, as the placenta mediates metabolic, gaseous, and nutritional exchanges between the maternal and fetal systems (Linck et al., 2024). Thus, phenotypic and reproductive divergences between breeds are often reflected in morphological variations of the uterine environment, potentially influencing fetal growth and survival during early gestation.

Based on these aspects, we hypothesize that genetic group differences impact uterine structure and fetal development. Therefore, this study compared the endometrial and placental morphology of Piau and Commercial sows at two gestational ages (25 and 35 days), aiming to identify structural evidence that may indicate how genetic differences influence uterine adaptation, placental development, and fetal viability.

## Methods

### Animals and experimental design

The experiment was conducted at the Universidade Federal de Viçosa, Brazil, following national animal welfare guidelines (CEUAP-UFV protocol 52-2024) and the European Union Directive 2010/63/EU on animal experimentation for the Piau genetic group. This study complied with the ARRIVE guidelines and Brazilian regulations on animal welfare. For the Commercial genetic group, the experimental protocol was approved by the Animal Research Ethics Committee of the Federal University of Viçosa (UFV), Minas Gerais, Brazil (protocol no. 06/2017), in accordance with the ethical principles in animal research established by CONCEA (2016).

A total of twelve sows were evaluated, divided equally into two genetic groups: six Piau and six Commercial sows. Each genetic group was further subdivided by gestational age, with three sows evaluated at 25 days of gestation and three sows evaluated at 35 days, resulting in n = 3 per group per gestational age. Estrus was synchronized using Regumate® (Merck Animal Health, USA) and artificial insemination was performed with semen from boars of the same genetic groups.

The Piau animals were sourced from the Swine Improvement Research and Extension Unit (UEPE), at the Universidade Federal de Viçosa UFV, where the research was conducted. The Commercial animals were also obtained from the same unit. The Commercial genetic group corresponds to a hybrid lineage (Large White × Landrace × Duroc) commonly used in Brazilian swine production.

### Sample collection and histological processing

At each gestational stage, females (n = 3 per genetic group per stage) were stunned (240V, 1.3A), and slaughtered. The uterus, ovaries, and conceptuses were collected, and phenotypic traits recorded: weight at slaughter (SW, kg), uterine weight (UW, kg), left uterine horn length (LUHL, cm), right uterine horn length (RUHL, cm), number of corpora lutea in the left ovary (NCLL), number of corpora lutea in the right ovary (NCLR), total number of corpora lutea (TCL), left ovary weight (LOW, g), right ovary weight (ROW, g), total ovarian weight (TOW, g), number of conceptuses (NC), number of viable conceptuses (NCV), and mortality rate (MR), calculated as: 100 - $\frac{\;Viable\;conceptus\;number}{Number\;of\;corpus\;luteum}X\;100$ (Costa et al., 2019).

Fetal weight (FW, g) was measured, and the coefficient of variation (CVc%), was calculated as:


$$\frac{Standard\;deviation\;of\;conceptuses\;weights}{Mean\;of\;conceptuses\;weights}X\;100$$ (1)

(Costa et al., 2019). Fetal cranio-caudal length (FLC, mm) was measured using a digital caliper (ZAAS Precision, Piracicaba, Brazil), following the methodology proposed by Guimarães et al. (2014).

**Figure 1:**
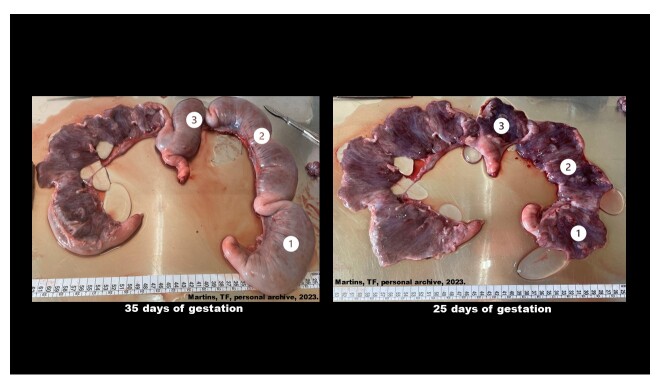
Representative structure of the uterine horns of a Piau sow at 25 and 35 days of gestation. Endometrial and placental samples were collected from three regions of the right uterine horn: (1) proximal, (2) medial, and (3) distal. This image is illustrative of a single animal and does not represent the average number of conceptuses described in the tables. Images of the Commercial genetic group were unavailable because samples were collected from archived material without photographic records at the time of collection.

For histomorphometric analysis, endometrial and placental samples were collected from three regions of the right uterine horn (proximal, medial and distal) of each genetic group and gestational stage (Figure 1). Samples were fixed in 4% paraformaldehyde, dehydrated, embedded in paraffin, and sectioned at 5 µm. Slides were stained with hematoxylin-eosin and mounted with Entellan (Merck, Germany) for microscopic evaluation. Endometrial and placental structures were assessed jointly, considering the type of lining epithelium, endometrial thickness, and the presence of uterine glands.

### Histomorphometric analysis

Endometrial and placental structures were assessed using an optical microscope (Olympus; BX53; Tokyo, Japan) equipped with 1.3MP CMOS digital camera BioCAM (Takachiho, Japan), and the TCapture program. Random images per tissue were analyzed for each sow using ImageJ software (version 1.50i; National Institutes of Health, Bethesda, MD, USA). The total number of blood vessels per area in the placental and endometrial tissues at both gestational ages was determined by counting and measuring the area of ​​each histological image.

Tissue volumetric proportions were determined using point-counting (266 points/image), evaluating placental tissues such as placental epithelial thickness (PET), placental connective tissue (PCT), placental vascularization (VB-P) and trophoblast epithelium (TRO). In this study, PET refers to the overall thickness of the placental layer visible in the histological section, measured perpendicularly from the fetal surface epithelium to the basal limit, encompassing the entire placental layer without detailing specific fetal membranes. TRO refers exclusively to the fetal trophoblastic epithelium, excluding the maternal uterine epithelium.

Although the uterine epithelium is visible in some histological sections, especially at 25 days of gestation, it was not quantified separately due to discontinuity, partial degeneration in some regions, and variability across samples. Thus, TRO represents only the fetal epithelial interface in direct contact with the maternal endometrium, while the maternal epithelium was excluded from volumetric analysis.

Considering endometrial uterine tissues: endometrial connective tissue (ECT), endometrial vascularization (VB-E) and uterine glands (EUG) were recorded (Tung et al., 2012). The percentage of points for the maternal and fetal portion, were calculated using the formula: volumetric proportion (%) = (Number of points in each structure) / (Total points in maternal or fetal tissue) ×100.

### Statistical analysis

Phenotypic data of sows and conceptuses, including SW, UW, LUHL, RUHL, NCLL, NCLR, TCL, LOW, ROW, TOW, NC, NCV, MR, CVc, FW and FLC were analyzed using R software. Differences between genetic groups and gestational ages were evaluated using ANOVA with mixed linear models, as described by the equation:


$$Y_{ijk}\text{​}=\;\mu \;+\;G_{i}\;+A_{j}\;+\;{\left(G\;\times \;A\right)}_{ij}\;+\;\varepsilon_{ijk\text{​}}$$ (2)

where: Y_ijk_ is dependent variable value for the *^i^*^-th genetic group,^ *^j^*^-th gestational age, and^ *^k^*^- th experimental unit;^ μ is the overall mean; G_i_ is the fixed effect of the *^i^*^-th^ genetic group (Piau or Commercial); A_j_​ is the fixed effect of the *^j^*^-th^ gestational age (25 or 35 days); (G × A)_ij_ is the interaction effect between the *^i^*^- th genetic group and the^ *^j^*^-th gestational age; and^ ɛ_ijk_ is the random error.

Normality was assessed using the Shapiro-Wilk test, and homogeneity of variances was evaluated using Levene’s test. Significance was set at *P* < 0.05, whereas values between 0.05 and 0.10 were considered trends. Logarithmic or square-root transformations were applied when necessary to stabilize variance and improve normality. For FW and FLC, wich did not meet ANOVA assumptions, even after transformation, the Kruskal-Wallis non-parametric test (Kruskal and Wallis, 1952) was applied (Tables 1 and 2, Supplementary Material).

Histomorphometric analyses of the placenta and endometrium were performed by calculating the percentage of each tissue type in relation to the total number of points per image, using the following formula:


$$\frac{Number\;of\;points\;in\;each\;structure}{Total\;points\;in\;maternal\;or\;fetal\;tissue\;}X\;100$$ (3)

(Souza et al., 2018).

For the histomorphometric data, the experimental unit was the individual sow. Although multiple tissue samples were collected from different regions, all samples from the same sow were considered subsamples within the experimental unit.

**Figure 2:**
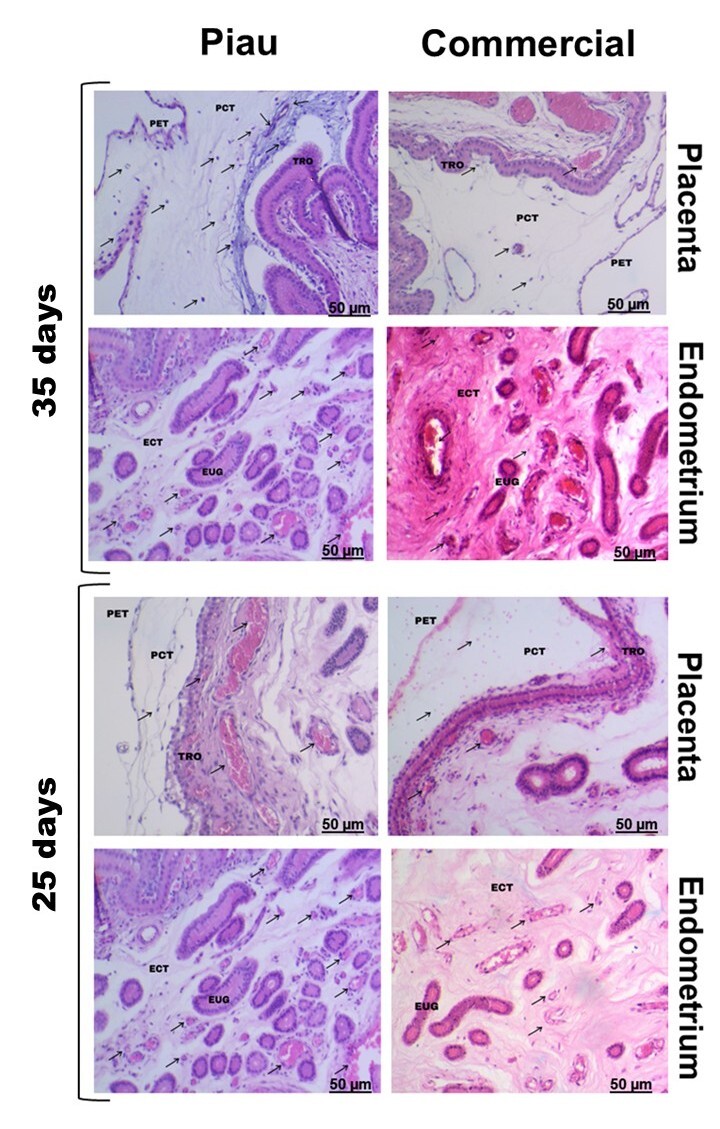
Histological images of placental and endometrial tissues of Piau and Commercial sows at 25 and 35 days of gestation. Placental components include PET (placental epithelial thickness), PCT (placental connective tissue), VB-P (placental vascularization), and TRO (fetal trophoblast epithelium, excluding maternal uterine epithelium). Endometrial components include ECT (endometrial connective tissue), VB-E (endometrial vascularization), and EUG (endometrial uterine glands). Note: In some 25-day images, the maternal uterine epithelium appears adjacent to the trophoblast but was not included in the quantitative analysis.

Linear mixed models were adjusted for each combination of genetic groups and gestational age, using the same model described above. Data were organized into subsets for placental tissues (PET, PCT, VB-P and TRO) and endometrial tissues (ECT, VB-E and EUG), allowing a detailed analysis of histomorphometric features in different genetic groups and gestational ages combinations (Figure 2).

## Results and discussion

The statistical analysis model included the interaction term between genetic group and gestational age. However, no significant interactions were detected (*P* > 0.10); therefore, only the main effects of genetic group and gestational age are presented and discussed.

### Phenotypic data of sows at 25 days of gestation

Significant phenotypic differences (*P* < 0.05) were observed between genetic groups at 25 days of gestation. Commercial sows showed higher values for SW (*P* < 0.0001), UW (*P* = 0.0045), LUHL (*P* = 0.0108), RUHL (*P* < 0.0001), NCLL (*P* = 0.0314), TCL (*P* = 0.0090), and TOW (*P* = 0.0248) compared to Piau sows (Table 1). A trend toward significance (*P* < 0.10) was observed for the NCLR (*P* = 0.05). No significant differences were found for LOW, ROW, NC, NCV, MR and CVc (Table 1).

**Table 1:** Phenotypic data for sows from the genetic groups Piau and Commercial in 25 days of gestation.

Traits	Piau (25 days)	Commercial (25 days)	SEM	P-value
SW (kg)	115.67	164.13	4.72	< 0.0001*
UW (kg)	1.28	2.90	0.21	0.0045*
LUHL (cm)	55.33	101.00	1.43	0.0108*
RUHL (cm)	55.00	117.43	3.67	<0.0001*
NCLL (count)	4.67	9.00	0.12	0.0314*
NCLR (count)	6.00	10.00	0.15	0.0500
TCL (count)	10.67	19.00	0.09	0.0090*
LOW (g)	4.83	8.19	0.19	0.1400
ROW (g)	6.90	8.68	0.79	0.4000
TOW (g)	11.73	16.87	1.32	0.0248*
NC (count)	11.33	17.00	0.33	0.2000
NCV (count)	10.67	16.67	0.00	0.2200
MR (%)	6.67	11.75	2.58	0.5500
CVc (%)	30.70	23.75	9.49	0.6900

Piau (25 days of gestation) and Commercial (25 days of gestation), with three sows per group (n = 3). SW: weight at slaughter; WW: uterine weight; LUHL: length of the left uterine horn; RUHL: right uterine horn length; NCLL: number of corpora lutea in the left ovary; NCLR: number of corpora lutea in the right ovary; TCL: total number of corpora lutea; LOW: left ovary weight; ROW: right ovary weight; TOW: total ovary weight; NC: number of conceptuses; NCV: number of viable conceptuses; MR: mortality rate; CVc: coefficient of variation among conceptuses; SEM: Standard error of the mean. *Significant at *P Anova* ≤ 0.05.

These results reflect the influence of genetic selection for reproductive efficiency in Commercial lines. A greater number of corpora lutea and longer uterine horns may favor embryonic maintenance and fetal development (Silva et al., 2014), mainly through enhanced uterine capacity and hormonal support during early pregnancy.

Moreover, previous studies suggest that the morphofunctional superiority of Commercial sows is associated with better uterine morphology and increased placental vascularization, improving placental efficiency and nutrient transfer to the conceptuses (Foxcroft et al., 2009). Such factors can enhance embryonic survival and promote more uniform development in the initial stages of pregnancy.

### Phenotypic data of sows at 35 days of gestation

At 35 days of gestation, this phenotypic superiority persisted, with significantly higher values (*P* < 0.05) for LUHL (*P* = 0.003), TCL(*P* < 0.0001), TOW (*P* = 0.0071), NC (*P* = 0.0226) , and NCV (*P* = 0.0198), in Commercial sows (Table 2). Trends toward significance were also observed for RUHL (*P* < 0.10), reinforcing the superior performance of Commercial sows.

**Table 2:** Phenotypic data of females and fetuses of Piau and Commercial genetic groups in 35 days of gestation.

Traits	Piau (35 days)	Commercial (35 days)	SEM	P-value
SW (kg)	130.23	161.87	5.51	0.0800
UW (kg)	3.03	5.48	0.57	0.0600
LUHL (cm)	94.67	143.83	4.83	0.003*
RUHL (cm)	92.67	155.83	14.1	0.0500
NCLL (count)	4.33	7.67	0.17	0.2900
NCLR (count)	7.33	11.33	0.40	0.1600
TCL (count)	11.67	19.00	0.67	< 0.0001*
LOW (g)	5.70	7.30	0.32	0.3100
ROW (g)	7.90	9.84	0.44	0.1300
TOW (g)	13.60	17.14	0.05	0.0071*
NC (count)	10.67	14.67	0.87	0.0226*
NCV (count)	10.33	13.67	0.55	0.0198*
MR (%)	10.72	28.07	2.07	0.1000
CVc (%)	17.55	11.08	3.09	0.3700
FW(g)	4.2	3.1	0.184	< 0.0001**
FLC (mm)	33	29.1	0.051	< 0.0001**

Piau (35 days of gestation) and Commercial (35 days of gestation), with three sows per group (n = 3). SW: weight at slaughter; WW: uterine weight; LUHL: length of the left uterine horn; RUHL: right uterine horn length; NCLL: number of corpora lutea in the left ovary; NCLR: number of corpora lutea in the right ovary; TCL: total number of corpora lutea; LOW: left ovary weight; ROW: right ovary weight; TOW: total ovary weight; NC: number of conceptuses; NCV: number of viable conceptuses; MR: mortality rate; CVc: coefficient of variation among conceptuses; FW: fetal weight; FLC: craniocaudal length; SEM: Standard error of the mean.*Significant at *P Anova* ≤ 0.05; ** For fetal traits (FW and FLC), values represent medians. P-values were obtained using the Kruskal–Wallis test.

This higher reproductive efficiency may be attributed to intensive genetic selection for growth and reproductive traits, a hallmark of intensive production systems (Silva et al., 2016). In contrast, Piau showed lower ovarian weight, fewer corpora lutea, and reduced uterine growth, which may compromise fetal viability.

However, Montes et al. (2018) suggest that local compensatory mechanisms, such as improved endometrial quality, maternal metabolic efficiency, and greater embryonic competence, may partially offset these structural limitations. Theses adaptations could maintain pregnancy even with lower reproductive investment.

Nonetheless, such constraints may still negatively impact fetal muscle development and overall productivity, especially considering that primary muscle fibers, crucial for determining postnatal fibers numbers, occur around 35 days of gestation (Wigmore and Stickland, 1983). Therefore, although Piau sows may rely on alternative physiological strategies to support gestation, their lower reproductive investment may ultimately result in reduced litter growth potential.

### Phenotypic data of the fetuses

**Figure 3:**
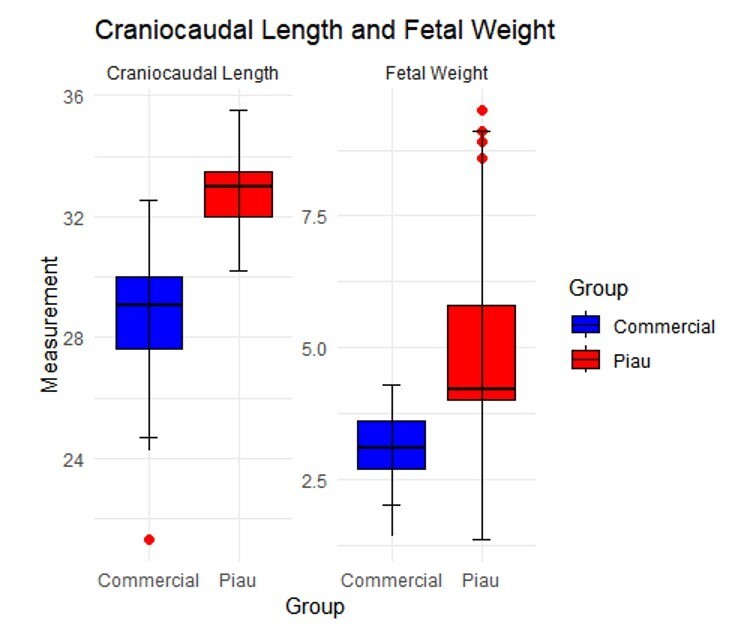
Boxplots for weight and craniocaudal length of fetuses at 35 days of gestation. The boxes represent the interquartile range (IQ ? R) with the median line, while the points outside the boxes indicate outliers. Statistical analysis was conducted to assess differences between treatments and/or groups.

Significant differences were observed between genetic groups at 35 days of gestation for FW and FLC, with Piau fetuses showing higher values ​​(*P* < 0.0001 for both traits) (Table 2; Figure 3). These results support previous evidence that Piau embryos exhibit accelerated growth during early gestation. Montes et al. (2018) reported that at 30 days, Piau embryos were longer than Commercial ones, but this difference disappeared by day 45, suggesting a limitation in sustaining growth throughout gestation.

This pattern may be linked to more intense early myogenesis in Piau fetuses, favoring primary muscle fiber formation (Sollero et al., 2011). In contrast, Commercial pigs seem to accelerate fetal growth later in gestational stages. Reis et al. (2016) detected increased expression of myogenesis-related genes, such as *MYOD1* and *MYOG*, at 40 and 70 days, indicating a delayed peak in muscle development. Similarly, Cagnazzo et al. (2006) reported that delayed myogenesis in Duroc pigs was associated with greater postnatal muscle hypertrophy, suggesting a similar developmental trajectory in Commercial pigs.

In addition, Brito et al. (2020) identified differences in apoptosis and myogenesis related gene expression. Commercial fetuses showed greater regulation of pro-apoptotic (*BAX*) genes at 30,45, and 60 days, suggesting a controlled mechanism of programmed cell death that supports secondary muscle fiber formation.

In contrast, Piau fetuses expressed these genes earlier, promoting primary fibers formation and increased cell survival during early development. Altogether, these results reveal distinct temporal patterns of fetal growth regulation and myogenesis between genetic groups. While Piau pigs prioritize early fetal growth, and myogenesis, Commercial pigs emphasize later muscle development, potentially influencing muscle fiber composition and postnatal meat quality.

### Histomorphometric analyses

At 25 days of gestation, significant differences (*P* < 0.05) were observed between the genetic groups. Commercial sows showed greater PET (*P* = 0.021), while Piau showed greater VB-P (*P* = 0.011) (Table 3). These results suggest different placental development strategies, with each genetic group prioritizing distinct aspects of the maternal-fetal interface.

**Table 3:** Comparison of the histomorphometric variables of uterine tissues of the right horns between Piau and Commercial sows at 25 and 35 days of gestation.

Organ	Piau (25 days)	Commercial (25 days)	SEM	P-value
PLACENTAL TISSUES				
PET (%)	5.67	8.98	0.06	0.021*
PCT (%)	71.12	69.57	0.18	0.705
VB-P (%)	11.65	6.45	0.08	0.011*
TRO (%)	11.56	15.01	0.07	0.068
ENDOMETRIAL TISSUES				
ECT (%)	62.85	82.36	0.12	< 0.0001*
VB-E (%)	18.97	8.60	0.23	< 0.0001*
EUG (%)	18.19	9.04	0.23	< 0.0001*
Organ	Piau (35 days)	Commercial (35 days)	SEM	P-value
PLACENTAL TISSUES				
PET (%)	6.18	4.21	0.01	0.08
PCT (%)	70.86	77.79	0.18	0.17
VB-P (%)	11.08	10.43	0.26	0.85
TRO (%)	11.88	7.56	0.07	0.06
ENDOMETRIAL TISSUES				
ECT (%)	58.92	71.33	0.24	0.001*
VB-E (%)	14.27	16.34	0.12	0.26
EUG (%)	26.81	12.34	0.45	< 0.0001*

Piau and Commercial genetic groups evaluated 25 days of gestation, with three sows per group and gestational age (n = 3). Placental tissues: PET: placental epithelial thickness; PCT: placental connective tissue; VB-P: placental vascularization; TRO: fetal trophoblast epithelium, excluding maternal uterine epithelium. Endometrial tissues: ECT: endometrial connective tissue; VB-E: endometrial vascularization; EUG = endometrial uterine glands. *Significant at *P Anova* ≤ 0.05. SEM: Standard error of the mean. Values are presented as percentages, totaling 100% per tissue group.

In the endometrium, Commercial sows displayed greater ECT (*P* < 0.0001), whereas Piau sows had greater VB-E (*P* = 0.0001) and greater EUG (*P* < 0.0001), suggesting a more vascularized uterine environment in the Piau, contrasting with the denser architecture in Commercial sows (Table 3).

Moreover, previous studies suggest that the morphofunctional superiority of Commercial sows related to better uterine morphology and placental efficiency (Foxcroft et al., 2009). Piau sows, with less selective pressure, may compensate through enhanced vascularization and glandular activity, optimizing the uterine environment even with lower ovulatory efficiency (Montes et al., 2018; Silva et al., 2014).

At 35 days of gestation, no significant differences (*P* > 0.05), were identified, but trends towards significance were observed for PET (*P* = 0.08) and TRO (*P* = 0.06) in Piau sows, suggesting an earlier onset of placental differentiation (Table 3). The absence of marked differences at 35 days may reflect physiological adaptations to the increasing fetal demand at this stage.

In the endometrium, greater ECT was observed in Commercial sows (*P* = 0.001), while Piau sows showed increased EUG (*P* < 0.0001) (Table 3). These findings indicate that each genetic group adopts distinct morphofunctional strategies during the initial stages of gestation.

These results align with the De Faria et al. (2019), who reported lower ovulation rate, fewer follicles, smaller follicular diameters, and lower estradiol levels in Piau compared to Commercial sows, along with differences in angiogenesis related gene expression. Such evidence indicates that genetic factors modulate reproductive physiology, reflecting divergent strategies: structural efficiency in Commercial sows versus vascular and functional efficiency in Piau.

Montes-Vergara et al. (2022) complement this interpretation, showing that although genes like *VEGFA, ANGPT1/2, TEK,* and *HIF1α* had similar expression between groups, endometrial vascular density was significantly greater in Piau sows at days 7 and 15 of gestation, indicating earlier vascular activation.

At 25 days, the predominance of blood vessels and connective tissue in the endometrium underscores their role in enhancing oxygenation, nutrient delivery, and waste removal. Vascular development, wich intensifies after implantation and peaks around 70 days, is essential for an efficient maternal-fetal interface (Reynolds et al*.,* 2006).

Therefore, early uterine structural differences may directly influence fetal nutrient transfer. In Commercial sows, the increase in ECT at 35 days, along with endometrial remodeling, reflects a more advanced adaptation to support fetal demands. These findings suggest that genetic groups employ distinct physiological strategies with potential consequences for gestational success and reproductive performance.

## Conclusion

Histomorphometric differences observed in the placenta and endometrium between the genetic groups directly influence gestational development. The Commercial group demonstrated greater reproductive investment, with more developed uterine horns and a higher number of corpora lutea, supporting sustained fetal development. In contrast, Piau sows, despite a lower ovulation rate, exhibited accelerated embryonic growth and higher placental vascularization at 25 days, which was not sustained at 35 days. These findings confirm the hypothesis that genetic groups differ in uterine and placental structure as well as fetal development, suggesting distinct morphofunctional strategies for supporting gestation. Further studies are needed to clarify how these structural differences affect fetal viability and overall reproductive efficiency.

## Supplementary Material

Supplementary material accompanies this paper.

Table 1 Normality (Shapiro–Wilk) and homogeneity of variances (Levene’s test) for raw data (pre-transformation) and transformed data (log or square-root) of the traits evaluated in Piau and Commercial genetic groups at 25 days of gestation.

Table 2 Normality (Shapiro–Wilk) and homogeneity of variances (Levene’s test) for raw data (pre-transformation) and transformed data (log or square-root) of the traits evaluated in Piau and Commercial genetic groups at 35 days of gestation.

This material is available as part of the online article from: https://doi.org/10.1590/1984-3143-AR2025-0070

## References

[B001] Almeida FRL, Alvarenga Dias ALN (2022). Pregnancy in pigs: the journey of early life. Domest Anim Endocrinol.

[B002] Brito EP, Reis EP, Penitente-Filho JM, Montes JC, Costa KA, Teixeira SA, Silva W, Pinho R, Guimarães JD, Costa EP, Lopes MS, Guimarães SEF (2020). Expression of apoptosis and myogenesis related genes during prenatal life in two divergent breeds of pigs. Theriogenology.

[B003] Cagnazzo M, Te Pas MFW, Priem J, De Wit AAC, Pool MH, Davoli R, Russo V (2006). Comparison of prenatal muscle tissue expression profiles of two pig breeds differing in muscle characteristics. J Anim Sci.

[B004] Costa KA, Saraiva A, Guimaraes JD, Marques DBD, Machado-Neves M, Barbosa LMR, Villadiego FAC, Veroneze R, Oliveira LF, Garcia IS, Teixeira SA, Guimarães SEF (2019). Dietary L-arginine supplementation during early gestation of gilts affects conceptuses development. Theriogenology.

[B005] De Faria VR, Pinho RO, Camilo BS, Guimarães JD, Silva FF, Lopes PS, Silva PV, Teixeira SA, Veroneze R, Penitente-Filho JM, Guimarães SEF (2019). Genes expression and phenotypic differences in corpus luteum and cumulus cells of commercial line and piau breed gilts. Theriogenology.

[B006] Foxcroft GR, Dixon WT, Dyck MK, Novak S, Harding JC, Almeida FC (2009). Prenatal programming of postnatal development in the pig. Soc Reprod Fertil Suppl.

[B007] Guimarães GC, Betarelli RP, Zangeronimo MG, Abreu ML, Almeida FR, Rosa MC, Ferreira LG, Alves LA, Assis CK, Lopes GC (2014). al. Vascularization of broad ligament of uterus and its relationship with fetal and placental development in gilts. Theriogenology.

[B008] Kridli RT, Khalaj K, Bidarimath M, Tayade C (2016). Placentation, maternal–fetal interface, and conceptuses loss in swine. Theriogenology.

[B009] Kruskal WH, Wallis WA (1952). Use of ranks in one-criterion variance analysis. J Am Stat Assoc.

[B010] Linck M, Tsoi S, Wenger II, Plastow GS, Dyck MK (2024). Placental transcriptome analysis in connection with low Litter Birth Weight Phenotype (LBWP) sows. Genes.

[B011] Montes JC, Penitente-Filho JM, Guimarães SEF, Lopes PS, Camilo BS, Shiomi HH, Lima DA, Pinho RO, Pereira JVTDN, Okano DS, Costa KA, Guimarães JD (2018). Aspects of sexual precocity and morphometry of uterus, placenta and embryos/fetuses in Piau breed and Commercial line gilts. Theriogenology.

[B012] Montes-Vergara JC, Penitente-Filho JM, Machado-Neves M, Machado LCM, Castaño-Villadiego FA, Costa KA, da Costa EP, de Campos CF, Ramírez-López CJ, Guimarães SEF, Lopes PS, Guimarães JD (2022). Influence of Genotype on Endometrial Angiogenesis during Early Pregnancy in Piau and Commercial Line Gilts. Animals.

[B013] Reis EPD, Paixão DM, Brustolini OJB, Silva FFE, Silva W, Araújo FMGD, Salim AC, Oliveira G, Guimarães SE (2016). Expression of myogenes in longissimus dorsi muscle during prenatal development in commercial and local Piau pigs. Genet Mol Biol.

[B014] Reynolds LP, Caton JS, Redmer DA, Grazul‐Bilska AT, Vonnahme KA, Borowicz PP, Luther JS, Wallace JM, Wu G, Spencer TE (2006). Evidence for altered placental blood flow and vascularity in compromised pregnancies. J Physiol.

[B015] Silva PV, Guimarães SEF, Guimarães JD, Nascimento CS, Lopes PS, Siqueira JB, Amorim LS, Fonseca E Silva F, Foxcroft GR (2014). Follicular dynamics and gene expression in granulosa cells, corpora lutea and oocytes from gilts of breeds with low and high ovulation rates. Reprod Fertil Dev.

[B016] Silva CLA, Van Den Brand H, Laurenssen BFA, Broekhuijse MJ, Knol EF, Kemp B, Soede NM (2016). Relationships between ovulation rate and embryonic and placental characteristics in multiparous sows at 35 days of pregnancy. Animal.

[B017] Stenhouse C, Hogg CO, Ashworth CJ (2018). Associations between fetal size, sex and both proliferation and apoptosis at the porcine feto-maternal interface. Placenta.

[B018] Sollero BP, Guimarães SEF, Rilington VD, Tempelman RJ, Raney NE, Steibel JP, Guimarães JD, Lopes PS, Lopes MS, Ernst CW (2011). Transcriptional profiling during foetal skeletal muscle development of Piau and Yorkshire–Landrace cross‐bred pigs. Anim Genet.

[B019] Souza ACF, Bastos DSS, Santos FC, Sertorio MN, Ervilha LOG, Gonçalves RV, Oliveira LL, Machado-Neves M (2018). Arsenic aggravates oxidative stress causing hepatic alterations and inflammation in diabetic rats. Life Sci.

[B020] Tung E, Roberts CT, Heinemann GK, De Blasio MJ, Kind KL, Van Wettere WH, Owens JA, Gatford KL (2012). Increased placental nutrient transporter expression at midgestation after maternal growth hormone treatment in pigs: a placental mechanism for increased fetal growth. Biol Reprod.

[B021] Wigmore PMC, Stickland NC (1983). Muscle development in large and small pig fetuses. J Anat.

