# Supplementary material for: Histomorphometric characterization of the uterus and placenta in Piau and Commercial sows during early gestation
Source: Anim Reprod. 2026 Jan 26;23(1):e20250070. doi: 10.1590/1984-3143-AR2025-0070 (PMC12970987; doi:10.1590/1984-3143-AR2025-0070)
Supplement: Table 1 [file 1984-3143-ar-23-1-e20250070-suppl1.pdf]

Supplementary Material

Table 1: Normality (Shapiro–Wilk) and homogeneity of variances (Levene's test) for raw data (pre-transformation) and transformed data (log or square-root) of the traits evaluated in Piau and Commercial genetic groups at 25 days of gestation.

Traits	*P*-value Shapiro-Wilk (pre)	*P*-value Levene (pre)	*P*-value Shapiro-Wilk (pos)	*P*-value Levene (pos)
SW (kg)	0.35	0.30	0.29	0.30
UW (kg)	0.68	0.33	0.83	0.46
LUHL (cm)	0.20	0.86	0.51	0.51
RUHL (cm)	0.90	0.29	0.52	0.19
NCLL (count)	8.18e-2*	1.00	0.60	0.78
NCLR (count)	0.25	0.79	0.85	0.98
TCL (count)	0.27	1.00	0.09	0.83
LOW (g)	2.16e-2*	0.86	0.13	0.67
ROW (g)	0.48	0.57	0.46	0.49
TOW (g)	0.69	0.37	0.61	0.33
NC (count)	3.13e-2*	0.79	0.18	0.85
NCV (count)	1.4 e-3*	1.00	0.27	0.77
MR (%)	0.05	0.75	0.33	0.91
CVc (%)	0.62	0.44	0.97	0.41

SW = weight at slaughter, UW= uterine weight, LUHL = length of the left uterine horn, RUHL = right uterine horn length, NCLL = number of corpora luteum in the left ovary, NCLR = number of corpora luteum in the right ovary, TCL = total number of corpus luteum, LOW = left ovary weight, ROW = right ovary weight, TOW = total weight of the ovaries, NC = number of conceptus, NCV = number of viable conceptuses, MR = mortality rate, CVc = coefficient of

variation among conceptuses. *Asterisks (*) indicate variables that did not meet the Shapiro–Wilk normality assumption in the raw (pre-transformation) data.*

Table 2: Normality (Shapiro–Wilk) and homogeneity of variances (Levene's test) for raw data (pre-transformation) and transformed data (log or square-root) of the traits evaluated in Piau and Commercial genetic groups at 35 days of gestation.

Traits	*P*-value Shapiro-Wilk (pre)	*P*-value Levene (pre)	*P*-value Shapiro-Wilk (pos)	*P*-value Levene (pos)
SW (kg)	0.96	0.45	0.84	0.59
UW (kg)	0.70	0.35	0.42	0.58
LUHL (cm)	0.79	0.37	0.83	0.51
RUHL (cm)	0.54	0.46	0.21	0.66
NCLL (count)	0.49	0.87	0.37	0.63
NCLR (count)	0.18*	0.85	0.85	0.95
TCL (count)	0.07*	0.37	0.20	0.37
LOW (g)	0.74	0.69	0.93	0.52
ROW (g)	0.37	0.59	0.47	0.51
TOW (g)	0.34	0.86	0.65	0.68
NC (count)	0.55	0.34	0.74	0.41
NCV (count)	0.85	0.37	0.81	0.47
MR (%)	0.58	0.65	0,62	0.26
CVc (%)	0.63	0.55	0.37	0.65
FW(g)	2.743e-6*	1.119e-11*	1.716 e-3**	9.117 e-5**
FLC (mm)	2.337e-4*	4.884 e-2*	4.493 e-6**	3.838 e-3**

SW = weight at slaughter, UW= uterine weight, LUHL = length of the left uterine horn, RUHL = right uterine horn length, NCLL = number of corpora luteum in the left ovary, NCLR = number of corpora luteum in the right ovary, TCL = total number of corpus luteum, LOW = left ovary

weight, ROW = right ovary weight, TOW = total weight of the ovaries, NC = number of conceptus, NCV = number of viable conceptuses, MR = mortality rate, CVc = coefficient of variation among conceptuses, FW = fetal weight, FLC = craniocaudal length of the fetuses. *Asterisks (**) indicate variables that did not meet the Shapiro–Wilk normality assumption in the raw (pre-transformation) data. Double *asterisks* (**) indicate variables for which the square-root transformation improved normality.*
